# Pulsatile ECMO: The Future of Mechanical Circulatory Support for Severe Cardiogenic Shock

**DOI:** 10.1016/j.jacbts.2024.02.015

**Published:** 2024-04-22

**Authors:** Douglas E. Vincent, Nader Moazami, David D’Alessandro, John F. Fraser, Silver Heinsar, Ellen T. Roche, Brian C. Ayers, Manisha Singh, Nina Langer, Shriprasad R. Deshpande, R.D.B. Jaquiss, Kiyotaka Fukamachi, Seyed Alireza Rabi, Asishana Osho, Taiyo Kuroda, Jamshid H. Karimov, Takuma Miyamoto, Palaniappan Sethu, Guruprasad A. Giridharan, Knut Kvernebo, Jack Copland

**Affiliations:** a VentriFlo, Inc, Pelham, New Hampshire, USA; b NYU Langone Medical Center, New York, New York, USA; c Massachusetts General Hospital, Boston, Massachusetts, USA; d Critical Care Research Group, The Prince Charles Hospital, University of Queensland, Brisbane, Queensland, Australia; e Massachusetts Institute of Technology, Cambridge, Massachusetts, USA; f Monash University, Melbourne, Victoria, Australia; g Children’s National Hospital & George Washington University, Washington, DC, USA; h Children’s Medical Centers/UT Southwestern Medical Center, Dallas, Texas, USA; i Cleveland Clinic, Learner Research Institute, Cleveland, Ohio, USA; j Thomas Jefferson Hospital, Philadelphia, Pennsylvania, USA; k University of Alabama-Birmingham, Birmingham, Alabama, USA; l University of Louisville, Louisville, Kentucky, USA; m University of Oslo, Oslo, Norway; n University of Arizona, Tucson, Arizona, USA

The utilization of temporary mechanical circulatory support (tMCS) has increased significantly over the last 40 years for stabilization of salvageable patients; however, there has not been much improvement in survival when used for cardiogenic shock, which has a current mortality of around 50%.^1^^,^^2^ Many efforts have been devoted to better understanding the stages of cardiogenic shock, as well as how combinations of both drugs and devices can increasingly be used to strive for recovery of the native heart.^3^^,^^4^ Despite the clear and urgent need for improving outcomes, none of the clinical trials has found convincing evidence for a survival advantage using tMCS compared with other forms of care.^1^^,^^3^ Therefore, new approaches to understanding the pathophysiology of cardiogenic shock, as well as novel technologies, would be beneficial.^2^

Veno-arterial extracorporeal membrane oxygenation (VA ECMO) is the most commonly used mode of tMCS for cardiogenic shock to efficiently provide cardiopulmonary support. Conventional VA ECMO uses continuous-flow devices to achieve circulatory support; this approach increases afterload and workload on a struggling heart, often leading to inability of the aortic valve to open and is associated with a high mortality rate even with restoration of adequate circulatory flow.^1^^,^^3^^,^^4^

The complication of increased left ventricular afterload has led to recognizing the importance of unloading or venting of the left ventricle (LV) while on ECMO to avoid distension and increased wall stress. This can be achieved by using various techniques, all of which require alternative technologies and major interventions and are sometimes deployed too late.^3^ At a physiological level, it is hypothesized that unloading the LV could help increase the likelihood of recovery by decreasing the heart’s metabolic demands and allowing time for it to rest.

A second limitation of the conventional continuous-flow ECMO systems is the resultant lack of pulsatility. Physiological pulsatile flow is important to deliver hemodynamic energy throughout the vascular bed and perfuse the organs at the microcirculatory level. To date, there has been less recognition and understanding of the disadvantages of lack of pulsatility in the systemic circulation, although awareness is growing.^2^

Early evidence suggests that pulsatile VA ECMO improves macrohemodynamics and helps the heart rest. It also preserves the microcirculatory hemodynamics and vascular adaptation throughout the systemic circulation, and protects multi-organ metabolic function, potentially leading to improved overall survival.^2^

We propose a novel volumetric-displacement ECMO device that overcomes these two major limitations of the current technologies.^5^ Our pump combines dynamic afterload reduction with generation of physiological pulsatile flow in a counter-pulsation mode. By triggering off the electrocardiogram signal, this pulsatile pump can deliver up to a 40 mL “stroke volume”^5^ during the native heart’s diastolic phase, against the closed aortic valve and into the elastic arterial vasculature. This technological advancement will more effectively unload the LV in favor of recovery while simultaneously providing a physiological pulse that will prevent the microcirculatory dysregulation throughout the body that often accompanies cardiogenic shock.

The system can deliver a true pulse through standard components, such as oxygenators, perfusion circuits, and cannulae, that are used clinically. It can be incorporated into typical VA ECMO configurations through various access sites, or in new methods of tMCS made possible by the unique pump design.

In the VA ECMO configuration, the device delivers an augmentative timed volumetric pulse into the systemic circulation. Benchtop testing shows a 10% to 20% reduction in the pressure-volume-area to native stroke-volume ratio, potentially resulting in a subsequent decrease in myocardial oxygen consumption while improving the total combined cardiac flow (augmentative extracorporeal flow + reduced native flow). Evidence suggests that this reduction in metabolic demand will help the heart rest and ultimately recover.^3^ Furthermore, with this pulsatility, greater hemodynamic energy is delivered throughout the systemic circulation.^2^

For patients who have isolated left-sided heart failure, the pump can be attached to a subclavian graft or femoral arterial cannula in a novel “arterial–arterial” configuration (AA-ECMO). In this setting, the pump can be timed to provide counter-pulsation regarding the native heart, thereby reducing the pressure and volume in the arterial tree ahead of left ventricular contraction and returning the volume (and pressure) to the arterial tree after the aortic valve has closed and the heart is in diastole. Not only does this reduce afterload and promote greater ejection fraction from the recovering LV, but the extracorporeal pump can also return the “stroke volume,” through the oxygenator, with greater hemodynamic energy, improving pulsatile perfusion to the heart and other downstream organs.

This configuration is only possible with a highly responsive, pressure-sensitive, volume-displacement or true pulse pump, one that can strategically remove a discrete volume of blood at the appropriate part of the native cardiac cycle and return it through an oxygenator (which is not possible with any other “unloading” technology). The design of this volume displacement pump uses a linear motor coupled to a translucent flexible membrane and clear chamber with separate one-way inlet and outlet valves,^4^ which when connected to a “Y” and the arterial graft or cannula, allow single-site access to the systemic circulation. In addition to being able to oxygenate the blood, nothing contacts any part of the heart. This method offers potential benefits, including reduced risk of bleeding, limb ischemia, LV collapse, arrhythmias, and device-made aortic regurgitation. It may also provide better multi-day management of an awake patient.

**Figure 1:**
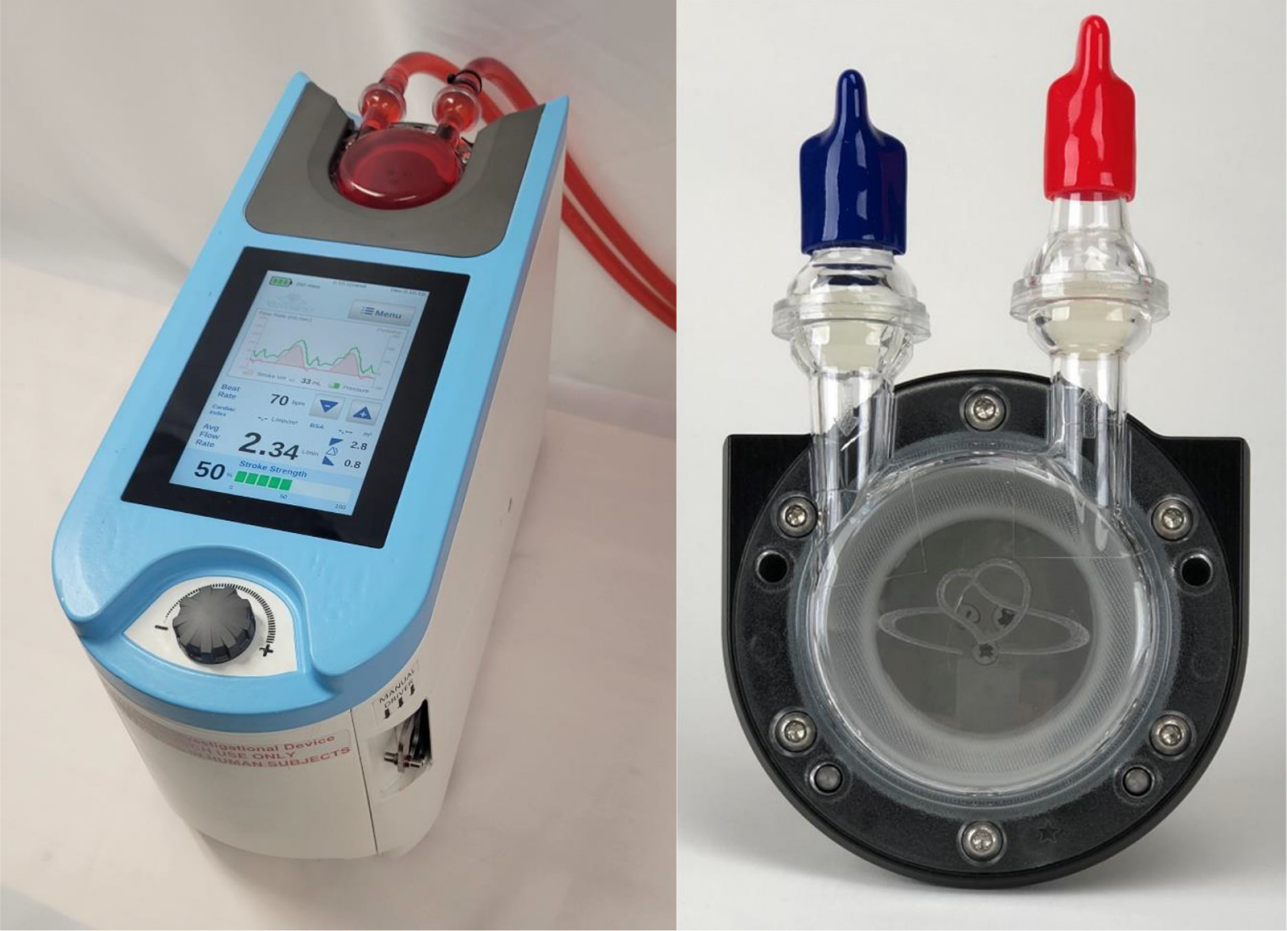
VentriFlo True Pulse Pump: Console and Blood Pump The technology described herein is covered by one or more patents issued or pending (see https://VentriFlo.com/Patents).

VentriFlo® True Pulse Pump™ is a patented pulsatile extracorporeal pump (Figure 1) that has been used in multiple extended (6-hour) cardiopulmonary bypass animal studies.^5^ This pump is incorporated into the novel ECMO circuits proposed here aimed at achieving the stated objectives of creating a timed pulse delivered into ECMO circuits that overcome the shortcomings of the conventional continuous-flow ECMO systems.
